# Comment on “Chinese Herbal Medicine for Osteoporosis: A Systematic Review of Randomized Controlled Trails”

**DOI:** 10.1155/2013/287176

**Published:** 2013-09-12

**Authors:** Qi-wei Lai

**Affiliations:** Department of Pharmacy, Guangdong Medical College, NO.1, Xincheng Dadao, Songshan Lake Science and Technology Industry Park, Dongguan, Guangdong Province, China

We read with interest the recent paper by Wang et al. [1]. Authors analyzed the results of the published literature where Chinese herbs were used as osteoporosis therapy (as measured by BMD). And authors concluded that Chinese herbs have merits in improving lumbar spine BMD as compared to the placebo or other standard antiosteoporotic drugs. 

However, there was a serious issue with this papaer. Due to significant differences existed among participants, study design, intervention, and outcome measurement, statistical heterogeneity was noted in the analysis of this study. According to the Cochrane Handbook for Systematic Reviews [2], *I* ^2^ ranges between 0% and 100%; *I* ^2^ values of 25%, 50%, and 75% are referred to as low, moderate, and high estimates. *I* ^2^ statistic greater than 50% suggested moderate heterogeneity, and a random effects model should be used. Instead, a fixed effects model was used for *I* ^2^ statistic less than 50%, which showed that heterogeneity could be neglected [2]. In the present study by Wang et al. [1], *I* ^2^ value was 94% in the analysis of Chinese herbs versus placebo on spine BMD; 96% in the analysis of Chinese herbs versus placebo on femoral neck BMD; 84% in the analysis of Chinese herbs versus standard antiosteoporotic drugs on lumber spine BMD; and 0% in the analysis of Chinese herbs versus standard antiosteoporotic drugs on the femoral neck BMD. But all the authors used fixed effects model regardless of the heterogeneity which was 0% or 96%. Is this reasonable?

 In my opinion, the present study by Wang et al. [1] gives us an important message: Chinese herb is effective in treatment of bone loss among patients with osteoporosis. Authors have done excellent job, and credit should be given to this work. But analysis method used in this study was irrational and should not be neglected, for this may influence the final results.
